# Nanoparticle Enhanced MRI Scanning to Detect Cellular Inflammation in Experimental Chronic Renal Allograft Rejection

**DOI:** 10.1155/2015/507909

**Published:** 2015-04-14

**Authors:** S. R. Alam, G. H. Tse, C. Stirrat, T. J. MacGillivray, R. J. Lennen, M. A. Jansen, D. E. Newby, L. Marson, P. A. Henriksen

**Affiliations:** ^1^Centre for Cardiovascular Science, The University of Edinburgh, Edinburgh EH16 4TJ, UK; ^2^MRC Centre for Inflammation Research, The University of Edinburgh, Edinburgh EH16 4TJ, UK; ^3^Clinical Research Imaging Centre, University of Edinburgh, Edinburgh EH16 4TJ, UK; ^4^Edinburgh Preclinical Imaging, University/BHF Centre for Cardiovascular Science, The University of Edinburgh, Edinburgh EH16 4TJ, UK; ^5^The Centre for Clinical Brain Sciences, The University of Edinburgh, Edinburgh EH16 4TJ, UK

## Abstract

*Objectives*. We investigated whether ultrasmall paramagnetic particles of iron oxide- (USPIO-) enhanced magnetic resonance imaging (MRI) can detect experimental chronic allograft damage in a murine renal allograft model.* Materials and Methods*. Two cohorts of mice underwent renal transplantation with either a syngeneic isograft or allograft kidney. MRI scanning was performed prior to and 48 hours after USPIO infusion using *T*2^∗^-weighted protocols. *R*2^∗^ values were calculated to indicate the degree of USPIO uptake. Native kidneys and skeletal muscle were imaged as reference tissues and renal explants analysed by histology and electron microscopy.* Results*. *R*2^∗^ values in the allograft group were higher compared to the isograft group when indexed to native kidney (median 1.24 (interquartile range: 1.12 to 1.36) versus 0.96 (0.92 to 1.04), *P* < 0.01). *R*2^∗^ values were also higher in the allograft transplant when indexed to skeletal muscle (6.24 (5.63 to 13.51)) compared to native kidney (2.91 (1.11 to 6.46) *P* < 0.05). Increased *R*2^∗^ signal in kidney allograft was associated with macrophage and iron staining on histology. USPIO were identified within tissue resident macrophages on electron microscopy.* Conclusion*. USPIO-enhanced MRI identifies macrophage.

## 1. Introduction

Chronic allograft damage (CAD), characterised by interstitial fibrosis and tubular atrophy (IFTA), is the commonest cause of transplant failure following surgery [1]. The demand for organ transplantation is expanding and waiting lists for a kidney are likely to increase in coming years [2]. Early identification of chronic allograft damage remains challenging but is crucial to allow intervention with immunosuppressive therapy. Renal biopsy remains the gold-standard for detecting allograft rejection but is associated with significant morbidity and mortality. The average complication rate is 7.4% with a life-threatening complication occurring in 1% [3, 4]. It would be advantageous to have a noninvasive imaging approach for the detection of acute rejection and IFTA. This would provide an alternative or adjunctive clinical assessment that may reduce the number of biopsies.

Current imaging techniques for monitoring allograft function involve the use of ultrasound to exclude ureteric obstruction or vascular compromise in the failing kidney. Measurement of vascular resistive index or the use of contrast-enhanced ultrasonography has been advocated but has not been clinically validated [5–7]. There is no imaging modality available to measure the development of graft fibrosis and current practice involves a biopsy when renal function deteriorates [8]. The role of monocytes and macrophages in chronic renal allograft damage has been well established [9]. Monocytes and macrophages are known to play a role in chronic renal allograft damage [10] and are key promoters of fibrosis in other organs, such as the liver [11, 12]. Several animal models of allograft rejection exhibit monocyte and macrophage infiltration in allograft tissue [13–17], and these cells have a central role in human chronic allograft damage [18, 19]. We have developed a model of chronic allograft damage: characterised by a single class II mismatch a kidney from C57BL/6^BM12^ (H-2B^BM12^) donor is transplanted into a C57BL/6 (H-2B) recipient and leads to the progressive development of interstitial fibrosis and tubular atrophy (IFTA) over 4 to 8 weeks. The key role of macrophages in this model has been demonstrated when transplants were performed into galectin-3 knockout recipients on a C57Bl/6 background. This led to an alteration in macrophage phenotype with reduced numbers of YM1-expressing macrophages in the knockout group and protection from IFTA [10].

Magnetic resonance imaging (MRI) offers detailed characterization of the kidney structure without using ionizing radiation and is suitable for monitoring renal allograft damage with repeated scanning. Iron oxide particles have been used as a contrast medium for MRI as they alter the *T*2^∗^ relaxation time of tissues in which they accumulate [20]. Ultrasmall (approximately 30 nm), superparamagnetic particles of iron oxide (USPIO) extravasate freely through capillaries and are taken up by tissue-resident inflammatory cells of the reticuloendothelial system [21]. Available USPIO include ferumoxytol (Rienso, Takeda; Feraheme, AMAG Pharmaceuticals), which is licensed for the treatment of anaemia caused by iron deficiency in patients with chronic kidney disease rather than as a contrast agent for MRI. Together with other groups, we have used USPIO as MRI contrast in clinical studies [20, 22–24].

Monocytes, macrophages, and to a lesser extent neutrophils take up USPIO, and accumulation in allograft rejection can be identified [25, 26]. MRI detected USPIO accumulation within the outer renal medulla in a model of renal ischaemia and this correlated histologically with USPIO uptake by macrophages [27]. USPIO have been used to investigate acute renal transplant rejection in preclinical models; however, these effects may have been due to ischaemia reperfusion injury [28, 29]. We hypothesized that they could be used to identify inflammation and fibrosis in a model of chronic renal allograft damage.

## 2. Materials and Methods

### 2.1. Murine Model of Renal Transplantation

Two cohorts of C57BL/6 mice underwent renal transplantation. Syngeneic renal transplants (*n* = 8) were performed between litter mates and allograft renal transplants from C57BL/6^BM12^ donors into C57Bl/6 recipients (*n* = 10). Characterised by a single class II mHC mismatch, such kidneys develop chronic allograft damage over a progressive twelve-week period. The model is not transplant-dependent as the contralateral kidney is left in situ. The isograft transplanted kidney and the native nontransplanted kidney were available as controls for comparison with the allograft kidney. Mice were bred in-house in the Biomedical Research Resources, University of Edinburgh, or purchased from Charles River. All animal experiments were performed under a project licence and in accordance with legislation in the Home Office Animal (Scientific Procedures) Act of 1986. Baseline MRI scanning was performed 4 weeks after transplant followed immediately by an infusion of USPIO by tail vein injection (4 mg/kg ferumoxytol; Rienso, Takeda). Repeat MRI scanning was performed 48 hours after infusion.

### 2.2. MR Imaging Protocols

All MRI experiments were performed using a 7-Tesla horizontal bore NMR spectrometer (Agilent Technologies, Yarnton, UK), equipped with a high-performance gradient insert (60-mm inner diameter), maximum gradient strength 1000 mT/m. Mice were anaesthetised with 1.5% isoflurane in oxygen/air (50/50, 1 L/min) and placed in a cradle (Rapid Biomedical GmbH, Rimpar, Germany). The rectal temperature and respiration rate were monitored throughout the experiments, and body temperature was maintained at 37°C with a heat fan. A 33-mm diameter birdcage volume coil (Rapid Biomedical GmbH, Rimpar, Germany) was used for radio frequency transmission and signal reception. For anatomical assessment and to aid placement of the slice for the *T*2^∗^ mapping sequence, respiration-gated *T*2-weighted fast spin echo images (echo train length of 8) of 1-mm slice thickness in a coronal orientation were collected with the following parameters: repetition time (TR) ≈ 3000 ms depending on the respiration rate; effective echo time = 36 ms; 16 slices, field of view = 35 mm × 35 mm; matrix = 192 × 128, 2 signal averages. For *T*2^∗^ mapping and calculation of *T*2^∗^ relaxation times, image acquisition used a gradient-echo, respiratory-gated pulse sequence (dummy pulses during respiratory movement) of 7 images weighted in *T*2^∗^ acquired consecutively: TE = 1.83, 3, 5, 7, 10, 12, and 15 ms and a TR of 60 ms. The field of view was 35 × 35 mm and the acquisition matrix 192 × 128 (in-plane resolution = 0.182 × 0.273 mm). Slice thickness was 1-mm with 2 signal averages.

USPIO imaging was performed with *T*2^∗^-weighted gradient-echo sequences using a 7 T MRI scanner. Quantitative analysis of USPIO accumulation was achieved by calculation of *T*2^∗^ relaxation times before and after administration of USPIO [20]. In order to optimise image analysis and prevent degradation due to “*T*2^∗^-blooming” artefacts, images were quantitatively analysed using a susceptibility gradient mapping postprocessing technique previously used in SPIO imaging to quantitate USPIO accumulation using changes in calculated *T*2^∗^ relaxation times [30].

### 2.3. Image Analysis

The seven echoes in the multiecho *T*2^∗^-weighted sequence were combined to generate a *T*2^∗^ map, in which the data represented the *T*2^∗^ value (*S*(*t*) = *S*(0)exp⁡(−*t*/*T*2^∗^)) for each voxel. This was achieved using in-house software developed in Matlab (Mathworks, USA). The *T*2^∗^ value is the decay constant for the exponential decay of signal intensity with time. In the presence of USPIO, the signal decays more rapidly due to local field inhomogeneities and the *T*2^∗^ value is reduced. By minimising the sum of the squares of errors between the data and an exponential function, the decay constant (i.e., *T*2^∗^ in ms) was obtained. An experimentally determined threshold for the coefficient of determination (*r* ^2^ > 0.85) was used to exclude data that did not have an acceptable exponential decay when SI was plotted against echo time. The inverse of *T*2^∗^, *R*2^∗^, was then calculated to assess USPIO uptake. The greater the accumulation of USPIO in tissues, the greater the *R*2^∗^ value.

*R*2^∗^ values were obtained from baseline and 48-hour scans using ANALYZE software (AnalyzeDirect Software, United States). Regions of interest were drawn on parenchyma, and pre-USPIO scans compared to post-USPIO scans. A semiquantitative analysis was made from the increase in *R*2^∗^ value. To correct for differences in blood pool USPIO concentration, due to infusion errors or difference in blood volume, the transplanted kidney *R*2^∗^ increase was indexed to the native kidney *R*2^∗^ increase. To provide a value of translational value to clinical medicine, where a normal healthy native kidney will not be present, the renal *R*2^∗^ increase was also indexed to skeletal muscle *R*2^∗^ increase.

### 2.4. Allograft Injury: Histology

Kidneys were divided and fixed fresh frozen or in methyl Carnoy's solution and embedded in paraffin. Tissue sections were stained with haematoxylin-eosin to allow histological analysis and reveal presence of inflammatory infiltrate [31, 32].

### 2.5. Allograft Injury: Cellular Infiltrate

Macrophage infiltration was identified by F4/80+ (Abcam, Cambridge, UK) staining by immunohistochemistry using paraffin embedded tissue sections. Light microscopy was performed and images were obtained and quantified by computer-assisted image analysis of 10 sequentially selected nonoverlapping fields of renal cortex and medulla and expressed as the percentage of tissue surface area positive for staining.

### 2.6. Electron Microscopy

For transmission electron microscopy, samples were fixed in 3% glutaraldehyde in 0.1 M sodium cacodylate buffer, pH 7.3, for 2 h and then postfixed in 1% osmium tetroxide in 0.1 M sodium cacodylate for 45 min. Samples were then dehydrated and embedded in araldite resin. Ultrathin 60-nm sections were cut from selected areas, stained in uranyl acetate and lead citrate and then viewed in a Philips CM120 transmission electron microscope, images obtained with a Gatan Orius CCD camera.

### 2.7. Statistical Analysis

Statistical analysis was performed with GraphPad Prism version 4.00 (GraphPad Software, San Diego, California, USA). Grafts were compared with native kidneys using paired two-tailed nonparametric *t*-tests (Wilcoxon matched-pairs signed rank test). Isograft and allograft kidneys were compared using nonpaired two-tailed nonparametric *t*-tests (Mann-Whitney test). Statistical significance was taken as a two-sided *P* < 0.05.

## 3. Results

One allograft and 2 isograft recipients sustained infarction of the transplanted kidney. This left 9 allograft mice and 6 isograft mice for analysis.

### 3.1. Change in *R*2^∗^ Values in Allograft and Isograft Kidneys

**Figure 1:**
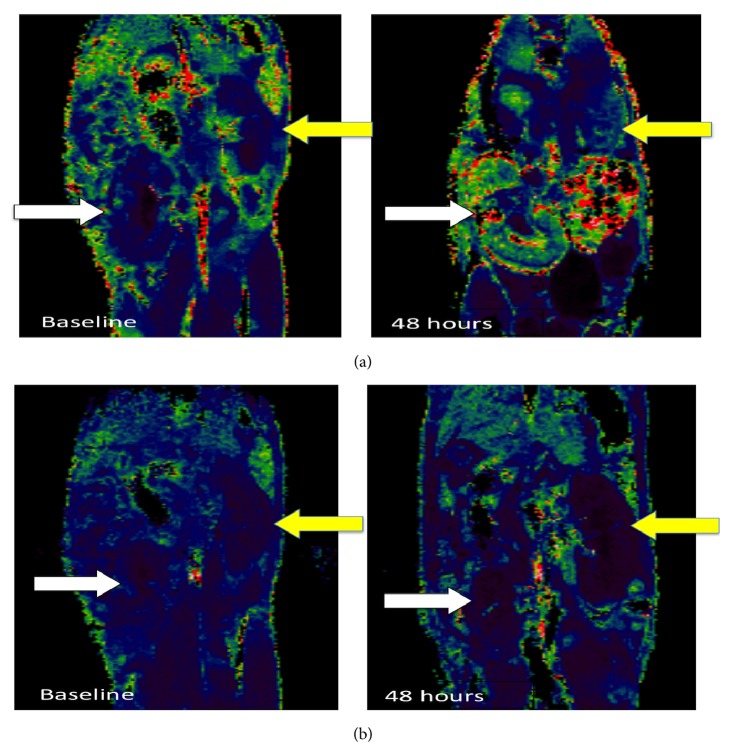
(a) Transplanted kidney (white arrows) compared to native kidney (yellow arrows) for allograft. (b) Transplanted kidney (white arrows) compared to native kidney (yellow arrows) for isograft.

Illustrative MRI scans with *R*2^∗^ signal derived colour maps of USPIO uptake in allograft and isograft kidneys are shown in Figures 1(a) and 1(b). Increased *R*2^∗^ signal and USPIO uptake are indicated by green and red colour.

Baseline *R*2^∗^ values were similar in native (median (interquartile range), 42.8 (38.5 to 50.5) ms^−1^) and allograft kidneys (44.2 (39.6 to 52.8) ms^−1^). USPIO administration increased *R*2^∗^ values in both isograft and allograft kidneys (Figure 1). The increase in *R*2^∗^ value at 48 h was greater in allograft kidneys (30.15 (14.0 to 68.0) ms^−1^) compared to native kidney (15.7 (4.5 to 26.8) ms^−1^), *P* < 0.01. In contrast, the increased *R*2^∗^ signal in isograft kidneys at 48 h (23.24 ms^−1^ (7.53 to 71.2) ms^−1^) was not different to native kidney (26.4 ms^−1^ (3.71 to 86.9) ms^−1^), *P* = 0.58.

**Figure 2:**
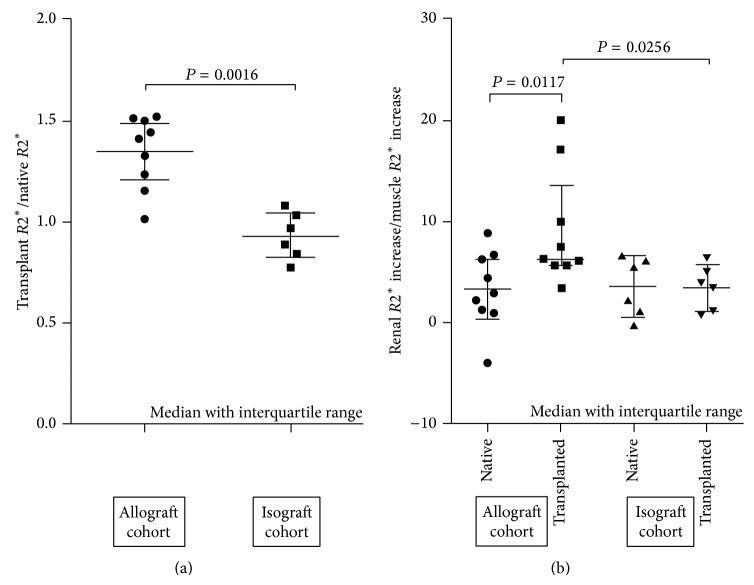
Increase in *R*2^∗^ value from baseline to 48 hours for transplanted kidney indexed to native kidney (a) and skeletal muscle (b).

*R*2^∗^ value increases indexed for changes in native kidney and skeletal muscle are shown in Figure 2. Median increase (interquartile range), indexed for native kidney, was greater in allografts 1.24 (1.12 to 1.36) compared to isografts 0.96 (0.92 to 1.04), *P* < 0.01, Figure 2(a). A similar result was obtained following indexing for skeletal muscle *R*2^∗^ increase (Figure 2(b)). The median increase in *R*2^∗^ value indexed to skeletal muscle was greater in the allograft kidney 6.24 (5.63 to 13.51) compared to native kidney 2.91 (1.11 to 6.46), *P* < 0.05. The corresponding skeletal muscle indexed signals were similar in isograft 3.83 (0.78 to 6.24) and native kidneys 3.63 (1.00 to 5.33) and significantly lower than the allograft signal, *P* < 0.05.

### 3.2. Histology and Electron Microscopy

**Figure 3:**
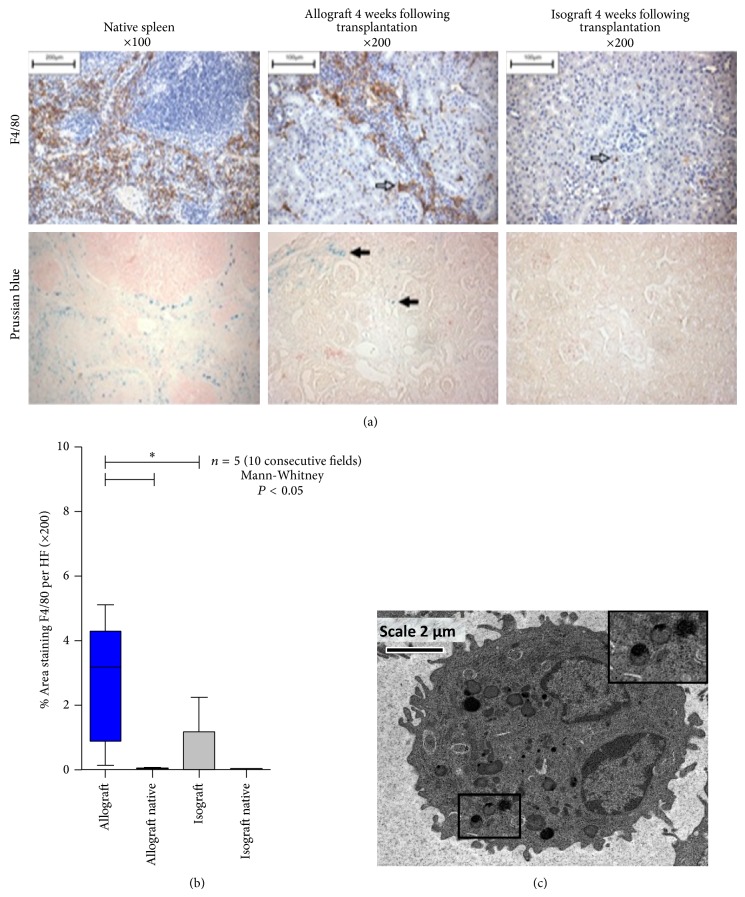
(a) F4/80 staining (top panel) for monocyte derived macrophages in the spleen and allograft and isograft (hollow arrows). Prussian blue staining (bottom panel) comparing iron deposition in the spleen, allograft tissue, and isograft (sold black arrows). (b) Histological monocyte count in allograft, isograft, and nontransplanted native kidneys. (c) Electron microscopy of macrophages in renal allograft tissue. The inlay (top right, magnification from black box) demonstrates USPIO within lysosomes.

F4/80 staining confirmed heavy macrophage resident cells in the allograft kidneys (2.70 ± 0.84 percentage area staining) with very few cells in the isograft kidneys (0.52 ± 0.44 percentage area staining) (Figures 3(a) and 3(b)). Iron staining with Prussian blue demonstrated deposition in allograft tissue with none in the isograft tissue. The reticuloendothelial tissue of the spleen was also rich in monocyte and iron staining. Electron microscopy confirmed monocyte/macrophage USPIO uptake within the renal tissue (Figure 3(c)).

## 4. Discussion

We have shown for the first time that USPIO-enhanced MRI can detect macrophage infiltration in a model of chronic inflammatory allograft damage. The prospect of noninvasive detection and monitoring of CAD, without resorting to renal biopsy, would be a significant advance in the management of renal transplant patients.

In this study the allograft USPIO signal was significantly increased compared to the native kidney. This direct comparison would be not feasible in a clinical study where the native kidney would be diseased or absent. Additionally human pathological factors may impact on the USPIO related signal. USPIO has a circulating half-life of 18–30 hours and persistence of particles in the circulation will affect the tissue *R*2^∗^ value due to perfusion [33]. In this study, differences in blood volume and organ perfusion, due to surgical blood loss or physiological variation related to inflammation and rejection, may contribute to the blood pool related signal in each animal. To address this problem, we indexed USPIO signal to skeletal muscle and native kidney. These findings on noninvasive imaging were associated with greatly increased macrophage infiltration and USPIO iron staining in allografts compared to isografts. We were able to further demonstrate macrophage USPIO uptake on scanning electron microscopy confirming that the USPIO signal on MRI was related to the macrophage infiltration in CAD.

It was not possible to be certain about the mechanism of cell labeling and distribution of USPIO within renal tissue. As a result of their smaller size, USPIO are less readily recognized by phagocytic cells and persist in the circulation for longer than other iron particles (plasma half-life 14–30 h in humans) [33, 34]. They are capable of passing through capillary walls, to be taken up through pinocytosis by tissue-resident macrophages and neutrophils [21, 25, 26]. Extravasated USPIO may remain in the interstitial space or be taken up by resident macrophages. Circulating USPIO may also be taken up by monocytes that subsequently infiltrate the kidney. These processes (monocyte uptake, USPIO blood extravasation, and resident macrophage uptake) may have different kinetics and could all contribute to the increased USPIO signal in allografts. The study aim was to provide proof of principle that allograft rejection can be detected noninvasively with contrast MRI but this approach could be used to further dissect out CAD mechanisms.

Significant advances have been made in the management of acute rejection as modern immunosuppressive agents target primarily T lymphocytes. However, the rate of CAD characterized by interstitial fibrosis remains relatively constant, giving rise to the loss of 4% renal transplants per year. Work from our own group has demonstrated that modification of macrophage biology can protect against fibrosis in this model of CAD and the role of macrophages in renal and liver fibrosis has been well established [10–12, 35]. Examination of macrophage phenotype was beyond the remit of this study, which focused primarily on imaging, but would be of interest to determine whether the macrophages were predominantly YM1-expressing profibrotic phenotype.

One limitation of the study is that imaging was performed at 4 weeks after transplant, when there was already histological evidence of fibrosis and, as the model is not transplant-dependent, it is not possible to ascertain whether deterioration in renal function had already occurred. However this is a proof of principle investigation to determine the feasibility of such an approach, and subsequent work will require imaging at earlier time points and correlating with renal function.

Blooming artefact associated with *T*2^∗^/*R*2^∗^ imaging with MRI is another potential limitation. These distortions can be erroneously included in the region of interest covering the renal tissue leading to falsely high *R*2^∗^ readings. In our study, this was particularly evident when the spleen laden with USPIO could distort the values in the neighbouring transplanted kidney. Care was taken to avoid drawing regions of interest over such areas. In addition, *T*2^∗^/*R*2^∗^ imaging identifies areas or tissue edema or hemorrhage in other organs, and as such the differing *R*2^∗^ values of baseline scans may have been due to differing amounts of edema [36]. Finally, the relationship between *R*2^∗^ value and iron accumulation is nonlinear, and so absolute increase in *R*2^∗^ value may not be directly proportional to increased inflammation [37]. The technique is semiquantitative and so increasing values do indicate an increase in the number of monocytes or macrophages in a tissue or an increase in activity. There was a range of values in the allograft group, suggesting a differing amount of inflammation in the different allografts. As the mechanism of rejection in this model of CAD is multifaceted a single measurement at 4 weeks is expected to have variation given that this model develops gradual histological injury up to 12 weeks and beyond [38].

The USPIO agent, ferumoxytol, is used as an intravenous iron supplementation agent for patients with end-stage renal failure. It has a good safety profile and is an ideal agent for investigation of transplant rejection in patients [39]. Further translational studies are needed to identify if there is a *R*2^∗^ threshold value which would identify a level of excessive inflammation requiring alteration of therapy.

In conclusion, we have developed an MRI technique for detecting inflammation in a model of chronic renal allograft damage. The protocol employs USPIO contrast that is compatible with patients who have renal dysfunction. This noninvasive approach for the detection of changes of CAD offers the possibility of avoiding renal biopsy in some patients. Translational studies are required to assess its applicability in clinical practice.

## Abbreviations

USPIO: Ultrasmall superparamagnetic particles of iron oxide
MRI: Magnetic resonance imaging
CAD: Chronic allograft damage.
